# Supplementary material for: RNAinsecta: A tool for prediction of precursor microRNA in insects and search for their target in the model organism Drosophila melanogaster
Source: PLoS One. 2023 Oct 9;18(10):e0287323. doi: 10.1371/journal.pone.0287323 (PMC10561860; doi:10.1371/journal.pone.0287323)
Supplement: S1 File — (DOCX) [file pone.0287323.s001.docx]

RNAinsecta: A tool for prediction of novel pre-microRNA in insects and search for their target in the model organism *Drosophila melanogaster*.

Adhiraj Nath^1^ and Utpal Bora^1*^

^1^ Department of BSBE, IIT Guwahati, North Guwahati, Assam, 784039, India

**Supplementary Material 1.**

**Insect pre-miRNA comparison with other organisms:**

We compared the pre-miRNA of insects with other organisms and groups of organisms such as Aves, Rice, Rodentia, Cattle and Humans. Different features such as GC content, Length, nucleotide counts, Loop length, Shannon entropy, etc. were calculated for each organism or group of organisms. Chi-square test was carried out between insects and the selected groups of organisms which was calculated by randomly taking 500 samples from each group.


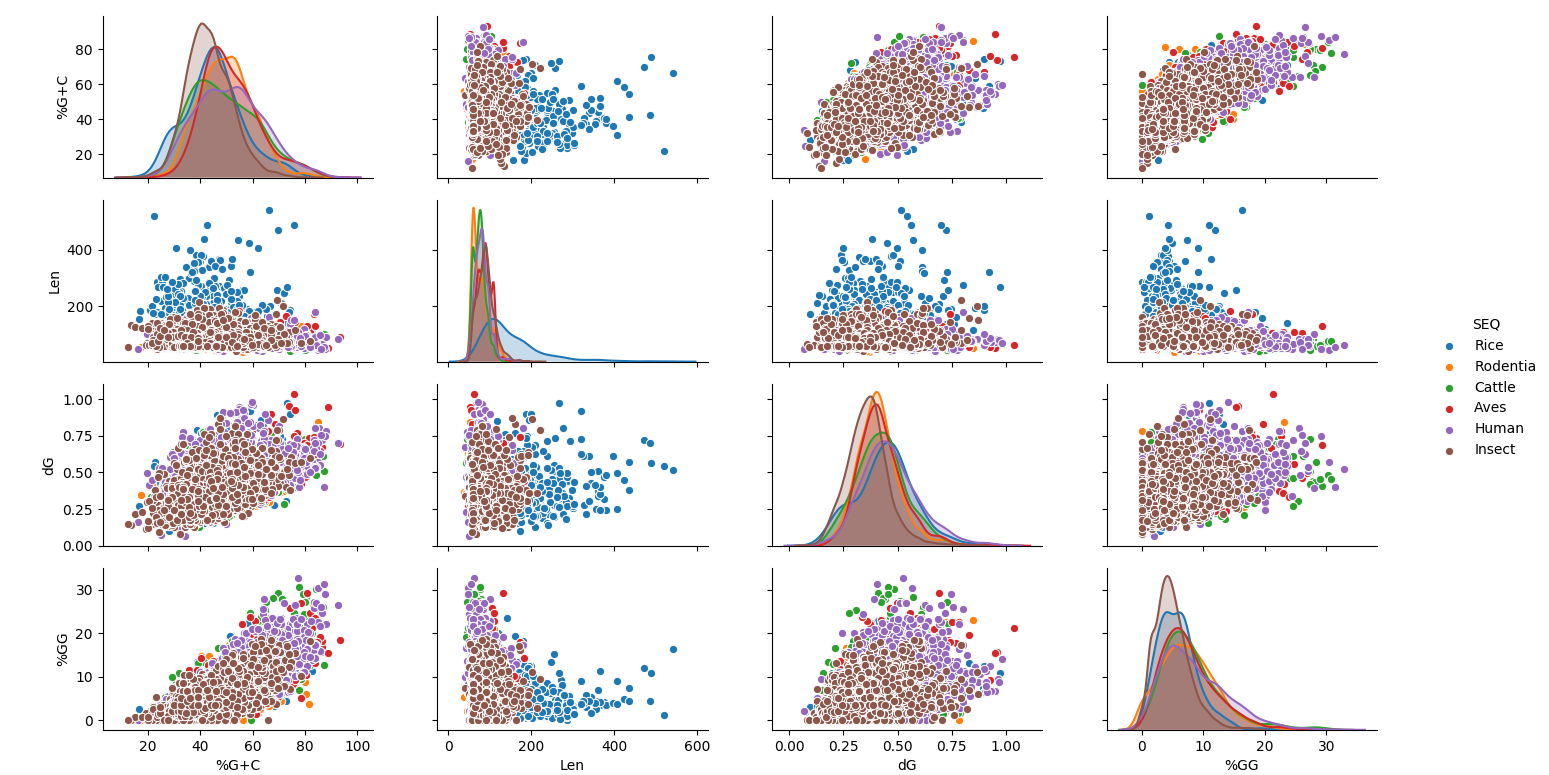
Pre-miRNA of insects differed from other organisms in many aspects such as length, MFE, Nucleotide counts, hairpin loop length, etc. upon which the previous tools were trained. Figure S1 shows the comparison between few parameters such as Length, %G+C, dQ and %GG that differ in insects from other organisms.

Figure S1: Comparison of insect pre-miRNA with other class of organisms. The comparation of various features of 500 randomly sampled pre-miRNA from Rice, Rodentia, Cattle, Aves, Human and Insects are is shown in the pair-plot scatter diagrams. Features such as GC percentage (%G+C), Length (Len), dG (MFE/Length) and dinucleotide percentage count of GG (%GG) were considered out of 61 features mentioned below. Multivariate gaussian distribution plot is given in the diagonals.

| **Table Title** | **Legend** |
| --- | --- |
| **1: Insect and Human pre-miRNA** | Chi-square test score and associated p-values of various parameters between insect and human. |
| **2: Insect and Rice pre-miRNA** | Chi-square test score and associated p-values of various parameters between insect and rice. |
| **3: Insect and Rodent pre-miRNA** | Chi-square test score and associated p-values of various parameters between insect and rodent. |
| **4: Insect and Cattle pre-miRNA** | Chi-square test score and associated p-values of various parameters between insect and cattle. |
| **5: Insect and Aves pre-miRNA** | Chi-square test score and associated p-values of various parameters between insect and aves. |

**Chi-square value of various parameters between insect and other organisms.**

Table 1-5 contains the chi-square test score and p-value for 61 features of pre-miRNA from insect and the other selected organisms. The *p-value* obtained from chi-square test suggested that insect pre-miRNA differs from Aves, Plants, Rodentia, Cattle and Humans. The %G+C of insect pre-miRNA is lowest among all the organisms that was compared. The length of pre-miRNA varied among different groups. The pre-miRNA of plants was found to be significantly longer (p-value < 0.01) than the other groups. Insects had shorter pre-miRNA than plants but longer than humans. The normalized MFE and Shannon entropy of insect pre-miRNA also varied from other groups of organisms.

1. **Insect and Human pre-miRNA:**

| **Sl. No.** | **Parameters** | **Chi-Square_Value** | **p-value** | **Sl. No.** | **Parameters** | **Chi-Square_Value** | **p-value** |
| --- | --- | --- | --- | --- | --- | --- | --- |
| 1 | Len | 918.3214141 | 1.02E-201 | 32 | %AG | 48.93155 | 2.65E-12 |
| 2 | A | 651.7312622 | 9.39E-144 | 33 | %AU | 562.6417 | 2.24E-124 |
| 3 | C | 9.838195879 | 0.001709251 | 34 | %CA | 3.254882 | 0.071211072 |
| 4 | G | 2.664868515 | 0.102586302 | 35 | %CC | 456.6692 | 2.55E-101 |
| 5 | U | 832.2972211 | 5.13E-183 | 36 | %CG | 139.0578 | 4.28E-32 |
| 6 | G+C | 11.12231632 | 0.000852952 | 37 | %CU | 63.34503 | 1.73E-15 |
| 7 | A+U | 1483.717795 | 0 | 38 | %GA | 2.564301 | 0.109300872 |
| 8 | AA | 170.4881999 | 5.79E-39 | 39 | %GC | 49.33994 | 2.15E-12 |
| 9 | AC | 67.15952315 | 2.50E-16 | 40 | %GG | 560.143 | 7.83E-124 |
| 10 | AG | 0.586444529 | 0.4437973 | 41 | %GU | 16.41303 | 5.09E-05 |
| 11 | AU | 827.9536001 | 4.51E-182 | 42 | %UA | 592.9923 | 5.60E-131 |
| 12 | CA | 26.75923317 | 2.30E-07 | 43 | %UC | 14.85345 | 0.000116195 |
| 13 | CC | 163.3947705 | 2.05E-37 | 44 | %UG | 28.23959 | 1.07E-07 |
| 14 | CG | 310.769907 | 1.48E-69 | 45 | %UU | 70.55004 | 4.49E-17 |
| 15 | CU | 0.036742262 | 0.847990749 | 46 | pb | 207.263 | 5.43E-47 |
| 16 | GA | 37.20174619 | 1.07E-09 | 47 | Npb | 0.122915 | 0.725894086 |
| 17 | GC | 0.890403448 | 0.345368001 | 48 | mfe | 3.836306 | 0.050153913 |
| 18 | GG | 189.1930252 | 4.77E-43 | 49 | dG | 2.977678 | 0.0844203 |
| 19 | GU | 125.0896939 | 4.86E-29 | 50 | Q | 89.08964 | 3.77E-21 |
| 20 | UA | 809.6357423 | 4.34E-178 | 51 | NQ | 0.127382 | 0.721161793 |
| 21 | UC | 108.3243821 | 2.28E-25 | 52 | D | 20.68904 | 5.40E-06 |
| 22 | UG | 12.46582748 | 0.000414465 | 53 | ND | 0.104828 | 0.746110736 |
| 23 | UU | 332.6416557 | 2.56E-74 | 54 | nstem | 843.8068 | 1.61E-185 |
| 24 | %A | 121.5987042 | 2.83E-28 | 55 | MFE1 | 0.001372 | 0.970449495 |
| 25 | %C | 132.2983058 | 1.29E-30 | 56 | MFE2 | 4.447836 | 0.034945495 |
| 26 | %G | 215.8669999 | 7.21E-49 | 57 | MFE3 | 3.289021 | 0.069744578 |
| 27 | %U | 187.7672144 | 9.77E-43 | 58 | MFE4 | 0.344789 | 0.557078147 |
| 28 | %G+C | 345.5943402 | 3.86E-77 | 59 | total_base | 207.263 | 5.43E-47 |
| 29 | %A+U | 308.212792 | 5.35E-69 | 60 | n_stems | 45.13261 | 1.84E-11 |
| 30 | %AA | 12.77394878 | 0.00035148 | 61 | avg_bp | 16.71504 | 4.34E-05 |
| 31 | %AC | 3.435804718 | 0.063797498 |  |  |  |  |

Table 1: Chi-square test score and associated p-values of various parameters between insect and human.

1. **Insect and Rice pre-miRNA:**

| **Sl. No.** | **Parameter** | **Chi-Square_Value** | **p-value** | **Sl. No.** | **Parameter** | **Chi-Square_Value** | **p-value** |
| --- | --- | --- | --- | --- | --- | --- | --- |
| 1 | Len | 8518.613758 | 0 | 31 | %AC | 18.98931805 | 1.31E-05 |
| 2 | A | 2307.229605 | 0 | 32 | %AG | 16.28015807 | 5.46E-05 |
| 3 | C | 2066.64927 | 0 | 33 | %AU | 4.771120517 | 0.028940984 |
| 4 | G | 2129.1967 | 0 | 34 | %CA | 1.143615812 | 0.284889583 |
| 5 | U | 2058.687084 | 0 | 35 | %CC | 104.7864948 | 1.36E-24 |
| 6 | G+C | 4194.450049 | 0 | 36 | %CG | 12.28226568 | 0.000457283 |
| 7 | A+U | 4348.107302 | 0 | 37 | %CU | 17.77485732 | 2.49E-05 |
| 8 | AA | 702.2932784 | 9.48E-155 | 38 | %GA | 35.85843939 | 2.12E-09 |
| 9 | AC | 260.5913467 | 1.28E-58 | 39 | %GC | 30.00024056 | 4.32E-08 |
| 10 | AG | 697.189871 | 1.22E-153 | 40 | %GG | 71.75141101 | 2.44E-17 |
| 11 | AU | 701.7485559 | 1.25E-154 | 41 | %GU | 73.63160367 | 9.41E-18 |
| 12 | CA | 573.9178457 | 7.89E-127 | 42 | %UA | 89.12702049 | 3.70E-21 |
| 13 | CC | 735.3842893 | 6.05E-162 | 43 | %UC | 1.112525873 | 0.291533538 |
| 14 | CG | 145.0698359 | 2.07E-33 | 44 | %UG | 0.472404924 | 0.491882867 |
| 15 | CU | 725.5236512 | 8.42E-160 | 45 | %UU | 69.72440372 | 6.82E-17 |
| 16 | GA | 805.0788354 | 4.24E-177 | 46 | pb | 3722.559444 | 0 |
| 17 | GC | 555.5655847 | 7.75E-123 | 47 | Npb | 0.388650958 | 0.533009369 |
| 18 | GG | 716.1834163 | 9.05E-158 | 48 | mfe | 6251.447599 | 0 |
| 19 | GU | 202.5474056 | 5.81E-46 | 49 | dG | 4.909641721 | 0.02670718 |
| 20 | UA | 309.8333754 | 2.37E-69 | 50 | Q | 2693.255826 | 0 |
| 21 | UC | 593.5032465 | 4.33E-131 | 51 | NQ | 2.424620481 | 0.119442166 |
| 22 | UG | 645.5352737 | 2.09E-142 | 52 | D | 839.7556706 | 1.23E-184 |
| 23 | UU | 541.5159737 | 8.82E-120 | 53 | ND | 0.613197441 | 0.433586451 |
| 24 | %A | 3.859700074 | 0.049459169 | 54 | nstem | 8906.705803 | 0 |
| 25 | %C | 36.04177866 | 1.93E-09 | 55 | MFE1 | 0.059657085 | 0.807038754 |
| 26 | %G | 27.36591139 | 1.68E-07 | 56 | MFE2 | 0.470563654 | 0.492727975 |
| 27 | %U | 61.05506349 | 5.55E-15 | 57 | MFE3 | 0.882888349 | 0.347411803 |
| 28 | %G+C | 62.83683004 | 2.25E-15 | 58 | MFE4 | 0.209444435 | 0.647203199 |
| 29 | %A+U | 50.0354342 | 1.51E-12 | 59 | total_base | 3722.559444 | 0 |
| 30 | %AA | 2.255848395 | 0.133110487 | 60 | n_stems | 274.1005785 | 1.45E-61 |
|  |  |  |  | 61 | avg_bp | 40.60739539 | 1.86E-10 |

Table 2: Chi-square test score and associated p-values of various parameters between insect and rice.

1. **Insect and Rodent pre-miRNA:**

| **Sl. No.** | **Parameters** | **Chi-Square_Value** | **p-value** | **Sl. No.** | **Parameters** | **Chi-Square_Value** | **p-value** |
| --- | --- | --- | --- | --- | --- | --- | --- |
| 1 | Len | 806.9565 | 1.66E-177 | 32 | %AG | 145.6287918 | 1.56E-33 |
| 2 | A | 651.7669 | 9.22E-144 | 33 | %AU | 420.0716086 | 2.35E-93 |
| 3 | C | 0.183035 | 0.668777571 | 34 | %CA | 62.7757414 | 2.32E-15 |
| 4 | G | 0.047975 | 0.826625541 | 35 | %CC | 360.601748 | 2.08E-80 |
| 5 | U | 890.1575 | 1.35E-195 | 36 | %CG | 467.0227331 | 1.42E-103 |
| 6 | G+C | 0.017765 | 0.893968877 | 37 | %CU | 202.9734995 | 4.69E-46 |
| 7 | A+U | 1540.251 | 0 | 38 | %GA | 1.527130271 | 0.216543651 |
| 8 | AA | 670.0491 | 9.75E-148 | 39 | %GC | 95.36211331 | 1.59E-22 |
| 9 | AC | 24.91625 | 5.99E-07 | 40 | %GG | 405.6588057 | 3.23E-90 |
| 10 | AG | 18.99368 | 1.31E-05 | 41 | %GU | 0.835827547 | 0.360592828 |
| 11 | AU | 651.2702 | 1.18E-143 | 42 | %UA | 303.4151586 | 5.94E-68 |
| 12 | CA | 0.7839 | 0.375950971 | 43 | %UC | 1.700764543 | 0.192188022 |
| 13 | CC | 118.9652 | 1.07E-27 | 44 | %UG | 105.2366888 | 1.08E-24 |
| 14 | CG | 719.9764 | 1.35E-158 | 45 | %UU | 673.5873469 | 1.66E-148 |
| 15 | CU | 34.42928 | 4.42E-09 | 46 | pb | 240.9011341 | 2.50E-54 |
| 16 | GA | 34.36372 | 4.57E-09 | 47 | Npb | 0.006534785 | 0.935570776 |
| 17 | GC | 3.277291 | 0.070244805 | 48 | mfe | 17.6357033 | 2.68E-05 |
| 18 | GG | 127.9262 | 1.16E-29 | 49 | dG | 2.021552023 | 0.155080563 |
| 19 | GU | 61.58661 | 4.24E-15 | 50 | Q | 6.583205734 | 0.010294533 |
| 20 | UA | 492.7646 | 3.57E-109 | 51 | NQ | 0.53523388 | 0.464414701 |
| 21 | UC | 71.75707 | 2.43E-17 | 52 | D | 0.908900553 | 0.340406303 |
| 22 | UG | 2.983826 | 0.084100235 | 53 | ND | 0.252538598 | 0.615293218 |
| 23 | UU | 1092.409 | 1.47E-239 | 54 | nstem | 807.2602825 | 1.42E-177 |
| 24 | %A | 159.6652 | 1.34E-36 | 55 | MFE1 | 0.00373182 | 0.951288632 |
| 25 | %C | 198.4418 | 4.57E-45 | 56 | MFE2 | 2.58736077 | 0.107719695 |
| 26 | %G | 241.6033 | 1.76E-54 | 57 | MFE3 | 2.268204426 | 0.13205282 |
| 27 | %U | 249.856 | 2.79E-56 | 58 | MFE4 | 0.382868262 | 0.536072174 |
| 28 | %G+C | 439.9476 | 1.11E-97 | 59 | total_base | 240.9011341 | 2.50E-54 |
| 29 | %A+U | 407.7895 | 1.11E-90 | 60 | n_stems | 27.81702522 | 1.33E-07 |
| 30 | %AA | 374.0611 | 2.44E-83 | 61 | avg_bp | 0.126086186 | 0.722525039 |
| 31 | %AC | 1.449584 | 0.228594643 |  |  |  |  |

Table 3: Chi-square test score and associated p-values of various parameters between insect and rodent.

1. **Insect and Cattle pre-miRNA:**

| **Sl. No.** | **Parameter** | **Chi-Square_Value** | **p-value** | **Sl. No.** | **Parameter** | **Chi-Square_Value** | **p-value** |
| --- | --- | --- | --- | --- | --- | --- | --- |
| 1 | Len | 918.3214141 | 1.02E-201 | 32 | %AG | 48.93155 | 2.65E-12 |
| 2 | A | 651.7312622 | 9.39E-144 | 33 | %AU | 562.6417 | 2.24E-124 |
| 3 | C | 9.838195879 | 0.001709251 | 34 | %CA | 3.254882 | 0.071211072 |
| 4 | G | 2.664868515 | 0.102586302 | 35 | %CC | 456.6692 | 2.55E-101 |
| 5 | U | 832.2972211 | 5.13E-183 | 36 | %CG | 139.0578 | 4.28E-32 |
| 6 | G+C | 11.12231632 | 0.000852952 | 37 | %CU | 63.34503 | 1.73E-15 |
| 7 | A+U | 1483.717795 | 0 | 38 | %GA | 2.564301 | 0.109300872 |
| 8 | AA | 170.4881999 | 5.79E-39 | 39 | %GC | 49.33994 | 2.15E-12 |
| 9 | AC | 67.15952315 | 2.50E-16 | 40 | %GG | 560.143 | 7.83E-124 |
| 10 | AG | 0.586444529 | 0.4437973 | 41 | %GU | 16.41303 | 5.09E-05 |
| 11 | AU | 827.9536001 | 4.51E-182 | 42 | %UA | 592.9923 | 5.60E-131 |
| 12 | CA | 26.75923317 | 2.30E-07 | 43 | %UC | 14.85345 | 0.000116195 |
| 13 | CC | 163.3947705 | 2.05E-37 | 44 | %UG | 28.23959 | 1.07E-07 |
| 14 | CG | 310.769907 | 1.48E-69 | 45 | %UU | 70.55004 | 4.49E-17 |
| 15 | CU | 0.036742262 | 0.847990749 | 46 | pb | 207.263 | 5.43E-47 |
| 16 | GA | 37.20174619 | 1.07E-09 | 47 | Npb | 0.122915 | 0.725894086 |
| 17 | GC | 0.890403448 | 0.345368001 | 48 | mfe | 3.836306 | 0.050153913 |
| 18 | GG | 189.1930252 | 4.77E-43 | 49 | dG | 2.977678 | 0.0844203 |
| 19 | GU | 125.0896939 | 4.86E-29 | 50 | Q | 89.08964 | 3.77E-21 |
| 20 | UA | 809.6357423 | 4.34E-178 | 51 | NQ | 0.127382 | 0.721161793 |
| 21 | UC | 108.3243821 | 2.28E-25 | 52 | D | 20.68904 | 5.40E-06 |
| 22 | UG | 12.46582748 | 0.000414465 | 53 | ND | 0.104828 | 0.746110736 |
| 23 | UU | 332.6416557 | 2.56E-74 | 54 | nstem | 843.8068 | 1.61E-185 |
| 24 | %A | 121.5987042 | 2.83E-28 | 55 | MFE1 | 0.001372 | 0.970449495 |
| 25 | %C | 132.2983058 | 1.29E-30 | 56 | MFE2 | 4.447836 | 0.034945495 |
| 26 | %G | 215.8669999 | 7.21E-49 | 57 | MFE3 | 3.289021 | 0.069744578 |
| 27 | %U | 187.7672144 | 9.77E-43 | 58 | MFE4 | 0.344789 | 0.557078147 |
| 28 | %G+C | 345.5943402 | 3.86E-77 | 59 | total_base | 207.263 | 5.43E-47 |
| 29 | %A+U | 308.212792 | 5.35E-69 | 60 | n_stems | 45.13261 | 1.84E-11 |
| 30 | %AA | 12.77394878 | 0.00035148 | 61 | avg_bp | 16.71504 | 4.34E-05 |
| 31 | %AC | 3.435804718 | 0.063797498 |  |  |  |  |

Table 4: Chi-square test score and associated p-values of various parameters between insect and cattle.

1. **Insect and Aves pre-miRNA:**

| **Sl. No.** | **Parameters** | **Chi-Square_Value** | **p-value** | **Sl. No.** | **Parameters** | **Chi-Square_Value** | **p-value** |
| --- | --- | --- | --- | --- | --- | --- | --- |
| 1 | Len | 61.76627219 | 3.87E-15 | 32 | %AG | 144.4826 | 2.79E-33 |
| 2 | A | 381.6699529 | 5.39E-85 | 33 | %AU | 579.0832 | 5.94E-128 |
| 3 | C | 139.4930765 | 3.44E-32 | 34 | %CA | 53.37173 | 2.76E-13 |
| 4 | G | 187.4562286 | 1.14E-42 | 35 | %CC | 356.5039 | 1.63E-79 |
| 5 | U | 450.262974 | 6.32E-100 | 36 | %CG | 163.7894 | 1.68E-37 |
| 6 | G+C | 326.38943 | 5.88E-73 | 37 | %CU | 219.8872 | 9.57E-50 |
| 7 | A+U | 831.8917453 | 6.29E-183 | 38 | %GA | 0.475384 | 0.490520867 |
| 8 | AA | 443.1704029 | 2.21E-98 | 39 | %GC | 388.4987 | 1.76E-86 |
| 9 | AC | 23.22547528 | 1.44E-06 | 40 | %GG | 436.8518 | 5.24E-97 |
| 10 | AG | 95.0952701 | 1.81E-22 | 41 | %GU | 18.29667 | 1.89E-05 |
| 11 | AU | 584.4577038 | 4.02E-129 | 42 | %UA | 588.3108 | 5.84E-130 |
| 12 | CA | 26.69141691 | 2.39E-07 | 43 | %UC | 12.62372 | 0.000380883 |
| 13 | CC | 232.7605994 | 1.49E-52 | 44 | %UG | 124.1619 | 7.76E-29 |
| 14 | CG | 220.1993829 | 8.18E-50 | 45 | %UU | 643.0437 | 7.28E-142 |
| 15 | CU | 154.7027339 | 1.63E-35 | 46 | pb | 15.13706 | 1.00E-04 |
| 16 | GA | 0.7905063 | 0.373947076 | 47 | Npb | 0.002045 | 0.963934201 |
| 17 | GC | 251.5284852 | 1.21E-56 | 48 | mfe | 161.0373 | 6.71E-37 |
| 18 | GG | 295.5107366 | 3.13E-66 | 49 | dG | 3.31838 | 0.068509312 |
| 19 | GU | 34.12439035 | 5.17E-09 | 50 | Q | 0.874532 | 0.3497037 |
| 20 | UA | 602.3266215 | 5.22E-133 | 51 | NQ | 0.122628 | 0.726201607 |
| 21 | UC | 20.99850791 | 4.60E-06 | 52 | D | 0.065907 | 0.797392103 |
| 22 | UG | 71.37481663 | 2.95E-17 | 53 | ND | 0.078789 | 0.778945017 |
| 23 | UU | 681.8512523 | 2.64E-150 | 54 | nstem | 89.02687 | 3.89E-21 |
| 24 | %A | 275.6314329 | 6.72E-62 | 55 | MFE1 | 0.005797 | 0.939306717 |
| 25 | %C | 282.5181789 | 2.12E-63 | 56 | MFE2 | 0.886497 | 0.346428226 |
| 26 | %G | 359.6920352 | 3.29E-80 | 57 | MFE3 | 2.485008 | 0.114935762 |
| 27 | %U | 347.0449475 | 1.86E-77 | 58 | MFE4 | 0.16074 | 0.688475856 |
| 28 | %G+C | 641.7339162 | 1.40E-141 | 59 | total_base | 15.13706 | 1.00E-04 |
| 29 | %A+U | 622.6445572 | 1.99E-137 | 60 | n_stems | 1.38889 | 0.238592666 |
| 30 | %AA | 376.4379186 | 7.42E-84 | 61 | avg_bp | 0.518863 | 0.471326912 |
| 31 | %AC | 10.67161946 | 0.001087918 |  |  |  |  |

Table 5: Chi-square test score and associated p-values of various parameters between insect and aves.
